# Health-related quality of life in thrombocytopenic patients with chronic hepatitis C with or without cirrhosis in the ENABLE-1 and ENABLE-2 studies

**DOI:** 10.1186/s12955-016-0447-1

**Published:** 2016-03-22

**Authors:** Kelly M. Grotzinger, Zobair M. Younossi, Edoardo G. Giannini, Pei-Jer Chen, Regina Rendas-Baum, Dickens Theodore

**Affiliations:** GlaxoSmithKline, 1250 S. Collegeville Road, Collegeville, PA 19426 USA; Inova Fairfax Hospital, 3300 Gallows Road, Falls Church, VA 22042 USA; University of Genoa, Viale Benedetto XV, no.6, Genoa, 16132 Italy; National Taiwan University Hospital, No.1, Changde Street, Zhongzheng District, Taipei 10048 Taiwan; QualityMetric, 24 Albion Road, Lincoln, RI 02865 USA; Novartis Pharmaceuticals Corporation, 1 Health Plaza, East Hanover, NJ 07936 USA

**Keywords:** Eltrombopag, Hepatitis C virus, HRQoL, Peginterferon alfa-2a, Ribavirin, Virologic response

## Abstract

**Background:**

Despite changes in the treatment paradigm towards non-interferon-based therapies, interferon-based treatments are still used in some geographical regions for treating patients with hepatitis C virus (HCV) infection. Use of eltrombopag with interferon-based treatment for patients with thrombocytopenia and HCV was assessed in two similarly designed phase 3 trials (Eltrombopag to Initiate and Maintain Interferon Antiviral Treatment to Benefit Subjects With Hepatitis C-Related Liver Disease [ENABLE-1 and ENABLE-2]). These trials also aimed to determine whether response to antiviral therapy (e.g., sustained virologic response [SVR]) is associated with changes in health-related quality of life (HRQoL). This pooled, post-hoc analysis aimed to (1) determine whether or not specific aspects of clinical response to treatment (i.e., achieving SVR) are associated with a significant change in HRQoL, and (2) to determine the magnitude and direction of the association between important changes in HRQoL, clinical response to interferon-based therapy (e.g., SVR) and treatment (eltrombopag or placebo), and patient and disease attributes.

**Methods:**

The Short-Form 36 Health Survey version 2 and Chronic Liver Disease Questionnaire–Hepatitis C Virus version were administered at various time points during the studies. Results from both trials were pooled for the analyses. Logistic regression analysis was used to assess the influence of 5 clinical factors (SVR, early virologic response [EVR], genotype [2/3 vs. non-2/3], treatment [eltrombopag or placebo], and cumulative interferon dose), plus other factors including ethnicity, model of end-stage liver disease score, and platelets as predictors of meaningful changes in HRQoL.

**Results:**

Between antiviral therapy baseline and the end of the 24-week post-treatment follow-up, declines in HRQoL were smaller in eltrombopag-treated patients than in placebo-treated patients, but the differences were not statistically significant. Mean changes among patients achieving SVR and EVR were small in comparison to thresholds of minimally important changes. Logistic models did not confirm the strength of the 5 clinical factors as predictors of meaningful changes in HRQoL during antiviral therapy, with the exception of the interaction between SVR and EVR (*P* = 0.0009). Asian ethnicity had a consistent effect on HRQoL, with East Asian patients being more likely to experience deterioration in HRQoL compared with white and/or other non-East Asian patients.

**Conclusions:**

While on active antiviral therapy, declines in HRQoL were not statistically different for eltrombopag-treated patients versus placebo-treated patients, suggesting that eltrombopag neither worsened HRQoL nor mitigated the effects of antiviral therapy on HRQoL.

**Electronic supplementary material:**

The online version of this article (doi:10.1186/s12955-016-0447-1) contains supplementary material, which is available to authorized users.

## Background

Thrombocytopenia is a frequent complication in patients with chronic hepatitis C virus (HCV) infection and correlates with liver disease severity [1]. It is more severe in patients with advanced disease undergoing interferon (IFN)-based antiviral therapy (AVT) and can be attributed to antiviral infection-induced liver cirrhosis and the inhibitory effects of IFN on thrombopoietin-induced development of megakaryocytic progenitor cells [1–4].

Chronic HCV infection is associated with reduced health-related quality of life (HRQoL), having a greater impact in patients with more severe liver disease [5]. Although decreased HRQoL was documented during the course of therapy with pegylated IFN (PEG-IFN) and ribavirin, multiple studies showed that HRQoL is restored to pre-AVT levels following stopping of IFN-based AVT after achieving a sustained virologic response (SVR) [6–11].

Eltrombopag (Promacta, GlaxoSmithKline, Research Triangle Park, NC, USA) is an oral, nonpeptide thrombopoietin receptor agonist approved for the treatment of thrombocytopenia in patients with chronic HCV to allow the initiation and maintenance of IFN-based AVT based on the ENABLE-1 and ENABLE-2 trials (ClinicalTrials.gov identifiers: NCT00516321 and NCT00529568, respectively) [12]. The ENABLE trials included a difficult-to-treat group of thrombocytopenic HCV-infected patients otherwise ineligible for IFN-based AVT based on their platelet counts.

The pivotal ENABLE trials not only established the efficacy and safety of eltrombopag in patients with HCV-associated thrombocytopenia, but also assessed HRQoL through the Short-Form 36 Health Survey version 2 (SF-36v2) and Chronic Liver Disease Questionnaire–HCV version (CLDQ-HCV) [13–15]. Although effective, therapy with IFN and ribavirin has side effects such as flu-like symptoms, anemia, and emotional changes [16, 17] that affect HRQoL [5, 18]. Based on published reports documenting the negative effects of IFN-based AVT on HRQoL [19, 20], HRQoL in patients in the ENABLE studies was expected to decline during the 24–48 weeks of IFN therapy due to the cumulative nature of the effects of IFN, and to return to baseline levels at the 24-weeks post-treatment follow-up visit [19]. Several reports indicate that patients who achieve SVR have improved HRQoL compared to patients who do not achieve SVR [7–11, 21, 22]. This pooled, post-hoc analysis aimed to (1) determine whether or not specific aspects of clinical response to treatment, such as achieving a SVR, are associated with a significant change in HRQL, and (2) to determine the magnitude and direction of the association between important changes in HRQoL, clinical response to IFN-based AVT (e.g., SVR) and treatment (eltrombopag or placebo), and patient and disease attributes.

## Methods

### Study design

**Fig. 1:**
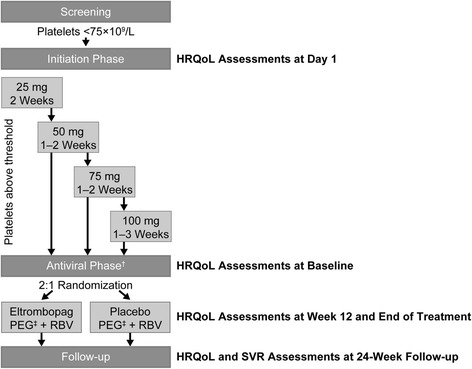
Design of the ENABLE trials* and timing of the SF-36v2 and CLDQ-HCV (HRQoL) assessments. *Each study included an open-label initiation phase, with patients receiving eltrombopag 25–100 mg for up to 9 weeks, with dose escalations being guided by platelet response. Patients whose platelet counts increased to a prespecified threshold to initiate AVT (ENABLE-1, 90 × 10^9^/L; ENABLE-2, 100 × 10^9^/L) were randomized 2:1 to receive either the same dose of eltrombopag they had received during the initiation phase or matching placebo. Hepatocellular carcinoma and portal vein thrombosis were assessed between screening and baseline and every 6 months thereafter through Doppler ultrasound of the abdomen. During the AVT phase, patients in ENABLE-1 were administered PEG-2a at 180 μg/week with RBV at 800 mg/day if they were infected with genotypes 2/3. If they were infected with genotypes other than 2/3, RBV doses were determined by body weight, with patients weighing <75 kg being given 1000 mg/day and patients weighing ≥75 kg being given 1200 mg/day. The weekly PEG-2b dose in ENABLE-2 was 1.5 μg/kg, while RBV doses were 800 mg/day, 1000 mg/day, 1200 mg/day, or 1400 mg/day based on body weights of ≤65 kg, 65–80 kg, 81–105 kg, or >105 kg, respectively. ^†^Twenty-four weeks for genotypes 2/3 and 48 weeks for other genotypes of hepatitis C virus. ^‡^PEG-2a in ENABLE-1 and PEG-2b in ENABLE-2. AVT, antiviral therapy; CLDQ-HCV, Chronic Liver Disease Questionnaire–Hepatitis C Virus version; HRQoL, health-related quality of life; PEG, pegylated interferon; RBV, ribavirin; SF-36v2, Short-Form 36 Health Survey version 2; SVR, sustained virologic response

The similarly designed ENABLE studies (Fig. 1) differed in the PEG-IFN used with oral ribavirin (ENABLE-1, PEG-2a; ENABLE-2, PEG-2b) and the platelet thresholds for initiating AVT (ENABLE-1, 90 × 10^9^/L; ENABLE-2, 100 × 10^9^/L) [12]. The protocols for both studies were reviewed and approved by the applicable ethics committee or institutional review boards at each center, in accordance with the International Conference on Harmonisation guidelines, applicable country-specific requirements, and the ethical principles of the 2008 Declaration of Helsinki. Written informed consent was obtained from all participants. Each study included a 2–9-week open-label phase during which the eltrombopag dose was titrated based on platelet response (Fig. 1). Patients not achieving platelet thresholds despite receiving eltrombopag 100 mg/day for 3 weeks entered the follow-up period. End of treatment was defined as the end of the planned AVT duration (24 weeks for genotypes 2/3, 48 weeks for non-genotypes 2/3, or withdrawal, whichever occurred first).

The American Association for the Study of Liver Diseases guidelines for stopping and futility were applied to PEG-IFN treatment [23]. Patients were stratified by HCV genotype (2/3 vs. non-2/3), baseline platelet counts (<50 × 10^9^/L vs. ≥50 × 10^9^/L), and HCV viral load (<800,000 vs. ≥800,000 IU/mL) at baseline [12]. All individuals involved in the study were blinded to treatment assignments.

PEG-IFN dose modifications were determined by local package instructions. The eltrombopag dose was reduced if the platelet count was >200 × 10^9^/L, and treatment interrupted and the dose reduced if the platelet count exceeded 400 × 10^9^/L.

**Fig. 2:**
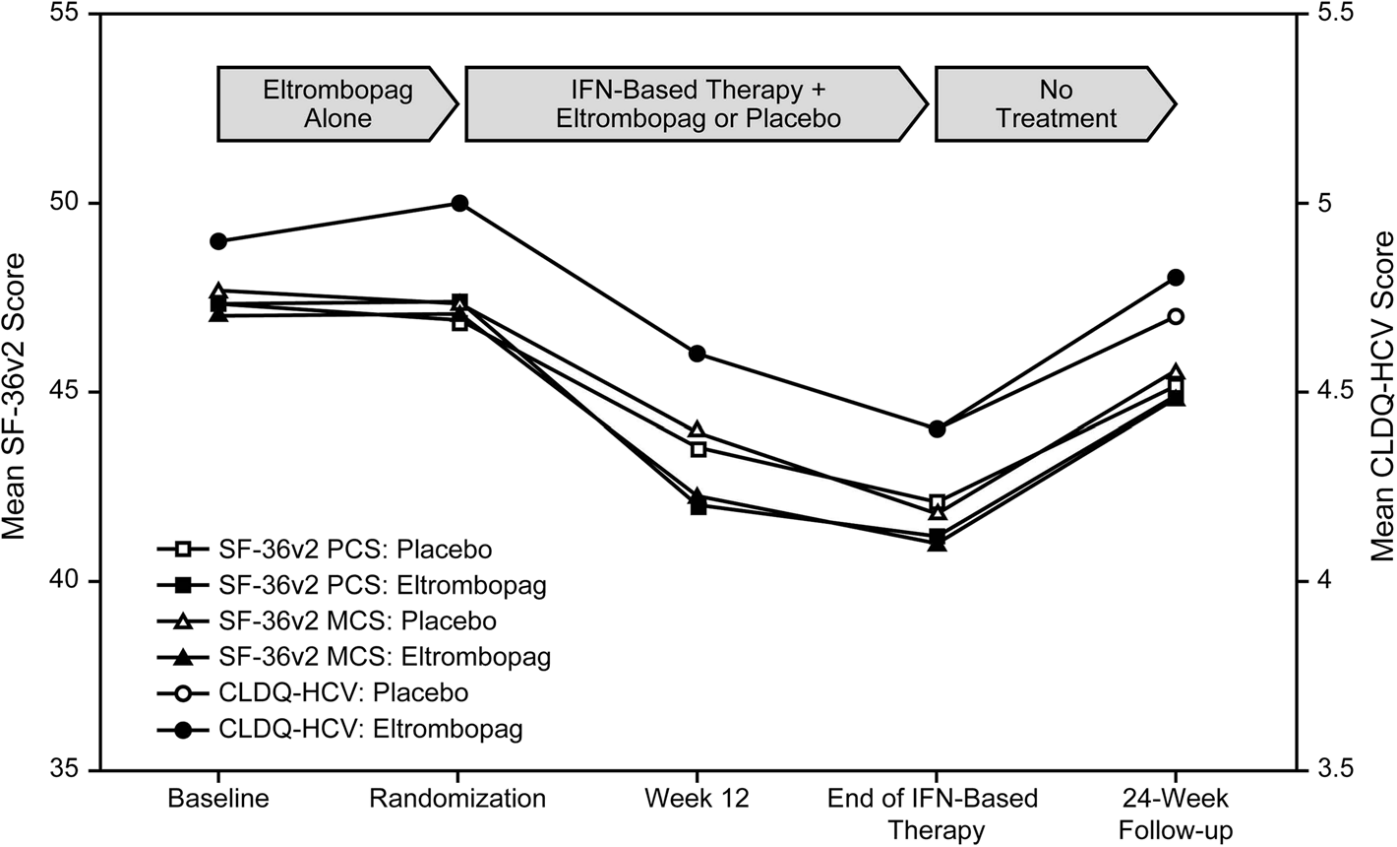
Mean values over time for SF-36v2 and CLDQ-HCV. CLDQ-HCV, Chronic Liver Disease Questionnaire–Hepatitis C Virus version; IFN, interferon; MCS, mental component summary; PCS, physical component summary; SF-36v2, Short-Form 36 Health Survey version 2

SVR was defined as continued non-detectable HCV RNA 24 weeks after completion of AVT (generally 48 or 72 weeks following initiation of AVT for genotypes 2/3 or at 72 weeks for non-genotype 2/3). EVR was defined as a reduction in HCV RNA (≥2 log_10_ drop or undetectable RNA) after 12 weeks of antiviral treatment. HRQoL was assessed at five time points during the ENABLE trials: baseline prior to receiving eltrombopag only (Eltrombopag Alone), at the time of randomization prior to INF-based therapy, Week 12, end of IFN-based therapy, and at the 24-week follow up visit (Fig. 2). However, only three HRQoL assessments are considered in this analysis; those done at the time of randomization, at the end of IFN-based therapy, and at the 24-week follow up visit, which corresponded to the final assessment of SVR. HRQoL was assessed prior to any interaction with physicians or staff, or release of information regarding viral load.

### Measures of HRQoL

This study used 2 patient-reported outcome instruments: SF-36v2 and CLDQ-HCV [6, 13].

#### Acute recall form of the SF-36v2

The SF-36 is a 36-item survey of self-reported functional health and well-being reported over 7 days [13]. Responses to 35 of the 36 items enable computation of a profile of functional health and well-being that consists of 8 subscales or HRQoL domains: physical functioning, role limitations due to physical health (role-physical), bodily pain, general health perceptions, vitality, social functioning, role limitations due to emotional problems (role-emotional), and mental health. Physical and mental health component summary (PCS and MCS, respectively) scores are then computed using the 8 subscales, to provide a broader metric of physical and mental HRQoL. The technique of norm-based scoring (NBS) is used to calculate the subscales and composite scores; this technique transforms or standardizes scale and component scores using the means and standard deviations (SDs) from a general US population normative sample (QualityMetric 2009 PRO Norming Study, QualityMetric, Lincoln, RI, USA; Additional file 1), drawn from a national probability sample of US non-institutionalized adults (KnowledgePanel, GfK Custom Research North America, New York, NY, USA). The NBS in the general US population is set to a mean ± SD of 50 ± 10 (i.e., T scores). Consequently, all scores <50 can be interpreted as being below, and scores >50 can be interpreted as being above, the general US population norm. All scales and component scores with NBS are scored on a common metric and facilitate meaningful comparisons. For more information on the calculation of NBS scale scores, see the Additional file 1 [13, 24].

Scores for the SF-36v2 PCS and MCS were derived according to the algorithm described by Farivar and colleagues [24] (Additional file 1). This algorithm differs from the original SF-36v2 with regard to summary score calculation in that it uses a correlated (oblique) physical and mental health factor model in lieu of the uncorrelated (orthogonal) factor solution used in the original SF-36v2.

The SF-36v2 has been used previously among patients with HCV and advanced fibrosis, had good measurement properties, and was responsive to change over time within patient populations without cirrhosis and with less severe liver disease [10, 25]. The literature suggests the SF-36v2 is capable of differentiating between patients with chronic HCV who do and do not achieve SVR. Those achieving SVR report better HRQoL, especially on role-physical and general health domains [7–11, 21, 22]. Generally, chronic HCV infection has a pronounced impact on the vitality, general health, physical function, and social function dimensions of the SF-36 [5, 9].

#### CLDQ-HCV

The CLDQ-HCV, which is based on the CLDQ [6, 15], is a 29-item patient self-reported questionnaire developed to measure HRQoL among patients with chronic liver disease and HCV. Approximately one-half of the questions in the CLDQ and the CLDQ-HCV are common. The remaining questions focus on symptoms and issues unique to HCV. The CLDQ-HCV was designed with a 2-week recall period, and items fall into 4 domains: activity/energy, emotion, systemic symptoms, and worry. Each item uses 7-point Likert scales ranging from 1 (all the time) to 7 (none of the time).

Responses on the CLDQ-HCV were coded into domain scores according to the algorithms designed by the developers; greater scores indicate better HRQoL. This permits decrements in HRQoL from baseline to be captured as negative values, while improved status is captured as positive values. Domain scores were calculated by averaging the responses within each domain, with the responses ranging from 1 to 7. An overall score was calculated by averaging scores across the 4 domains.

### Statistical analysis

Differences between groups for normally distributed variables were evaluated and tested for statistical significance using analysis of variance (ANOVA) or analysis of covariance (ANCOVA) models. When comparing means among three or more groups, if significant differences were observed from omnibus ANOVA or ANCOVA models, post-hoc pair-wise comparison of means were computed using Tukey’s test to control for alpha inflation. The Chi-Square and Fisher’s exact tests were used to assess statistical significance related to contingency tables.

Differences between groups were interpreted using Cohen’s d [26]. For analyses of change scores, Cohen’s d was calculated as the ratio of the difference in mean change scores between the eltrombopag and placebo groups versus the pooled standard deviation of the changes scores.

#### Models of clinical factors associated with important changes in HRQoL

The association between important changes in HRQoL and 5 clinical factors (SVR, EVR, genotype [2/3 vs. non-2/3], treatment [eltrombopag vs. placebo], and cumulative dose of IFN) was evaluated using logistic regression models with the following independent variables: the 5 clinical factors, age (<40 vs. ≥40 years), sex, ethnicity (East Asian, other Asian, white, and other), baseline Model for End-Stage Liver Disease (MELD) score (<10 vs. ≥10), baseline platelet count (<50 × 10^9^/L vs. ≥50 × 10^9^/L), and interaction terms between SVR and EVR, treatment and EVR, and genotype and cumulative IFN dose. Two dependent variables were evaluated for each HRQoL scale (8 subscales and 2 summary measures of the SF-36v2 [PCS and MCS] and 4 subscales and overall score of the CLDQ-HCV). “Drop in HRQoL score” was defined as a drop in HRQoL greater in magnitude than the minimally important change (MIC) during active AVT (end of treatment – AVT baseline). “Return to baseline score” was defined as a 24-week post-treatment follow-up HRQoL score within the MIC of the AVT baseline.

The MIC for the CLDQ-HCV was defined as one-half of the SD of the AVT baseline score [27]: overall HRQoL = 0.6; energy/activity = 4.0; emotion = 4.8; systemic = 3.8; and worry = 5.4. For SF-36v2 scores derived using NBS, the recommended MIC values were used to evaluate important changes: 3.4, 4.6, 4.3, 3.4, 6.2, 7.2, 6.2, 6.9, 4.5, and 6.2, for PCS, MCS, physical functioning, role-physical, bodily pain, general health perceptions, social functioning, role-emotional, and mental health scores, respectively [13]. Each patient in the ENABLE pooled sample was classified as experiencing a meaningful change if the difference between his or her baseline and post-baseline scores was greater in absolute value than the MIC.

The predictive powers of both SVR and EVR were modeled because both characterize response to therapy. Other considerations that underlie the model include that duration of AVT depends upon genotype [28] and that exposure for 80 % of the planned time and at 80 % of the planned dose of IFN is associated with the greatest likelihood of sustained viral response [29].

The model used to estimate the log odds of each HRQoL outcome is as follows:

$$ \begin{array}{l} \log \left(\frac{\mathrm{P}\left[\mathrm{HRQoL}\ \mathrm{outcome}=1\right]}{\mathrm{P}\left[\mathrm{HRQoL}\ \mathrm{outcome}=0\right]}\right)=\alpha +{\beta}_1SVR+{\beta}_2EVR+{\beta}_3Gen2,3+{\beta}_4 Eltrombopag+{\beta}_5IFN+\hfill \\ {}{\beta}_6\left( Age<40\right)+{\beta}_7 Male+{\beta}_8\left( East\  Asian\right) + {\beta}_9\left( Other\  Asian\right) + {\beta}_{10} MELD\left(<10\right)+{\beta}_{11}\left( Plat<50\right)\hfill \\ {}\kern6em +{\beta}_{12}EVR*SVR+{\beta}_{13} Eltrombopag*IFN+{\beta}_{14}Gen2,3*IFN\hfill \end{array} $$

Model assumptions were evaluated for lack of fit and evidence of multicollinearity. The assumption of linearity between continuous variables and the logit of the outcome was examined using the Hosmer-Lemeshow test [30]. Excessive collinearity or other indications of model misfit or violation of model assumptions led to modifications to the proposed model specifications.

#### Hierarchy of significance testing for HRQoL scales

The PCS and MCS of the SF36v2 and the overall HRQoL as derived from the CLDQ-HCV were modeled first, followed by the subscales representing challenges to physical health and functioning. The subscales or domains capturing mental and emotional health were modeled last. Table 1 shows the hierarchical order for testing of the null hypotheses.

**Table 1:** Hierarchical order for testing of the null hypotheses

	SF-36v2	CLDQ-HCV
Aggregate summary scores		
	Physical functioning (PCS scores)	Overall HRQoL (average of the 4 domain scores of the CLDQ-HCV)
	Mental well-being (MCS scores)	
Physical health and functioning domain scores		
	Physical functioning	Systemic symptoms
	Role-physical	Activity/energy
	Bodily pain	
	Vitality	
Mental and emotional health scores		
	Mental health (SF-36v2 subscale)	Emotion
	General health (SF-36v2 subscale)	Worry
	Role-emotional (SF-36v2 subscale)	
	Social functioning (SF-36v2 subscale)	

*CLDQ-HCV* chronic liver disease questionnaire–hepatitis C virus version, *HRQoL* health-related quality of life, *MCS*, mental component summary, *PCS*, physical component summary, *SF-36v2* short-form 36 health survey version 2

Results are presented as odds ratios (ORs; with confidence intervals) for each HRQoL outcome. Model parameters provide the OR of the HRQoL event (drop during treatment or return to baseline level at the 24-week post-treatment follow-up assessment) for the groups represented by that factor (e.g., patients who achieved SVR vs. those who did not). For the cumulative IFN dose—a continuous variable—the interpretation of the OR refers to the effect of a 1-point difference in the cumulative IFN dose.

#### Multiplicity adjustments and hypotheses tested

Two families of hypotheses (Specifications A and B) were defined as follows: the odds of restoring HRQoL at the Week 24 follow-up visit (Specification A) or the odds of a drop in HRQoL greater than the MIC during active AVT (Specification B), where HRQoL is measured as scores on the SF-36v2 and CLDQ-HCV. Specification A tests whether the odds of restoring HRQoL at the 24-week post-treatment follow-up visit are significantly greater for patients with one of the 5 clinical factor characteristics (e.g., SVR) compared to patients without that characteristic (e.g., non-SVR). Specification B tests whether the odds of a drop in HRQoL greater than the MIC during active AVT are significantly greater among patients with one of the 5 clinical factor characteristics (e.g., SVR) compared to patients without that characteristic (e.g., non-SVR).

Fifteen tests were conducted for each specification, with 1 test conducted per HRQoL domain or summary score defined previously. Adjustment for multiplicity was explored. Model specifications were fitted for the purpose of answering the research questions and reflect underlying data structure assumptions, goodness-of-fit statistics, and embedded adjustments for multiple tests.

The study also examined logistic regression models (data not shown), which permitted the exclusion of particular clinical variables and/or interactions among the variables (not reported) reflecting *a priori* concerns of multicollinearity among clinical predictors, particularly the relationship between SVR and EVR, as well as *a posteriori* considerations. Type 3 analysis of effects was used to determine *P* values for each variable included in the model using SAS/STAT(R) version 9.22 (SAS, Cary, NC, USA).

## Results

Baseline demographic and clinical characteristics of the combined group of patients from the ENABLE-1 and ENABLE-2 trials (*n* = 1441) are shown in Table 2. At baseline, mean SF-36v2 acute PCS and MCS scores (submission scoring) of the pooled patient population were 47.1 and 47.0, respectively, while respective median PCS and MCS scores were 48.6 and 48.3. The mean (median) overall CLDQ-HCV scores of the pooled population were 4.9 (5.0). Mean (median) scores on the individual components of the CLDQ-HCV were: activity, 29.7 (31.0); emotion, 44.3 (46.0); systemic symptoms, 29.1 (30.0); and worry, 38.9 (40.0).

**Table 2:** Baseline demographic and clinical characteristics

Characteristic	ENABLE-1 and −2 Pooled (*N* = 1441)
Age (years)	
Mean	52.1
Median	52.0
SD	8.6
Baseline platelet count (×10^9^/L)	
Mean (SD)	56.9 (13.4)
Median	59.5
AVT baseline HCV RNA value transformed by log_10_, mean (SD)	5.7 (0.8)
Age, n (%)	
≥ 40 years	1338 (92.9)
< 40 years	103 (7.2)
Bridging fibrosis/cirrhosis	
Patients with measurements, n	1268
*n* (%)	1143 (90.1)
Ethnicity, *n* (%)	
White/Other	1109 (77.0)
Other Asians	186 (12.9)
East Asians	146 (10.1)
Actual HCV genotype, *n* (%)	
Non-2/3 or missing	995 (69.0)
2/3	446 (31.0)
Body mass index, *n* (%)	
< 30 kg/m^2^	1028 (71.3)
≥ 30 kg/m^2^	406 (28.2)
Missing	7 (0.5)
Depression in past medical history	17 (1.2)

*AVT*, antiviral therapy, *HCV* hepatitis C virus, *SD* standard deviation

### Changes in HRQoL scores across treatment and viral response groups

Changes in mean scores for the SF-36v2 and CLDQ-HCV over the trial period are shown in Fig. 2. The mean change in scores between Week 24 and AVT baseline ranged between −2 and −3, indicating that SF-36v2 and CLDQ-HCV scores were lower at Week 24 than at baseline. There were small differences between the eltrombopag and placebo groups. The small group differences between Week 24 and AVT baseline, and the lack of statistical significance related to treatment in the ENABLE studies, may be partially because the number of observations was too small to make a robust estimate.

Mean changes in HRQoL scores between the end of treatment (24 or 48 weeks, depending on the genotype) and baseline are shown in Table 3 [24]. Achievement of either EVR or SVR did not explain the changes in HRQoL. There was a slight decline in SF-36v2 and CLDQ-HCV scores between AVT baseline and 24 weeks post-treatment among patients who did and did not achieve EVR (data not shown).

**Table 3:** Drop in SF-36 and CLDQ-HCV HRQoL scores from antiviral baseline to end of ATV treatment, and at the 24-week post-treatment follow-up by treatment group

	Drop in ATV BL to End of Treatment Mean (95 % CI)			Drop in ATV BL to 24-week FU Mean (95 % CI)		
	PBO	EPAG	Cohen’s d	PBO	EPAG	Cohen’s d
SF-36v2 acute						
Physical functioning	−5.2 (−6.1, −4.4)	4.8 (−5.4, −4.2)	0.05	−2.2 (−3.1, −1.4)	−1.6 (−2.2, −1.0)	0.08
Role-physical	−4.7 (−5.7, −3.8)	−5.5 (−6.2, −4.8)	−0.08	2.4 (−3.3, −1.4)	−2.2 (−2.9, −1.6)	0.02
Bodily pain	−4.6 (−5.6, −3.7)	−4.8 (−5.5, −4.1)	−0.02	2.3 (−3.4, −1.3)	−1.7 (−2.4, −1.0)	0.06
General health	−3.8 (−4.5, −3.0)	−2.9 (−3.4, −2.3)	0.11	2.3 (−3.1, −1.4)	−1.8 (−2.4, −1.2)	0.06
Vitality	−6.0 (−7.0, −5.1)	−6.1 (−6.7, −5.4)	−0.00	−2.1 (−3.0, −1.2)	−1.4 (−2.1, −0.8)	0.07
Social functioning	−5.3 (−6.3, −4.3)	−6.5 (−7.3, −5.8)	−0.12	2.5 (−3.5, −1.6),	−2.2 (−2.9, −1.5)	−0.12
Role-emotional	−5.3 (−6.5, −4.0)	−6.8 (−7.7, −5.8)	−0.11	−3.1 (−4.2, −2.0)	−2.8 (−3.7, −1.9)	0.03
Mental health	−5.8 (−6.7, −4.9)	−5.3 (−6.0, −4.6)	0.05	−2.1 (−3.0, −1.1)	−1.7 (−2.4, −1.1)	0.07
PCS^a^	−5.8 (−6.6, −5.0)	−6.2 (−6.8, −5.6)	−0.05	−2.8 (−3.6, −1.9)	−2.3 (−2.8, −1.7)	0.03
MCS^a^	−6.3 (−7.2, −5.4)	−6.4 (−7.1, −5.8)	−0.02	−2.6 (−3.5, −1.7)	−2.1 (−2.7, −1.5)	0.06
CLDQ-HCV						
Overall	−0.6 (−0.7, −0.5)	−0.6 (−0.6, −0.5)	0.02	−0.3 (−0.4, −0.2)	−0.2 (−0.3, −0.2)	0.06
Activity	−5.6 (−6.3, −4.8)	−5.6 (−6.2, −5.1)	−0.01	−1.9 (−2.7, −1.2)	−1.8 (−2.3, −1.3)	0.02
Emotion	−4.8 (−5.7, −3.8)	−4.5 (−5.1, −3.9)	0.03	−1.9 (−2.8, −1.0)	−1.8 (−2.4, −1.1)	0.02
Systemic symptoms	−4.0 (−4.6, −3.3)	−4.0 (−4.4, −3.5)	−0.00	−2.3 (−3.0, −1.6)	−1.7 (−2.2, −1.3)	0.09
Worry	−2.5 (−3.4, −1.6)	−1.8 (−2.4, −1.2)	0.07	−1.7 (−2.7, −0.7)	−0.9 (−1.6, −0.2)	0.08

*CI* confidence interval, *CLDQ-HCV* chronic liver disease questionnaire–hepatitis C virus version, *MCS* mental component summary, *PCS* physical component summary, *SF-36v2* short-form 36 health survey version 2

^a^Scored as a correlated physical and mental health factor model [24]

The association between EVR at 12 weeks post-AVT baseline and HRQoL change scores was weak, with SF-36v2 mean change scores that ranged from −6.5 to −3.9 among patients who did not achieve EVR and from −6.4 to −2.7 among those who achieved EVR. For the 5 CLDQ-HCV scales, mean change scores ranged from −5.8 to −0.7 among patients who did not achieve EVR and from −5.5 to −0.5 for those who achieved EVR at 12 weeks.

Declines in SF-36v2 and CLDQ-HCV scores among those who achieved SVR (SVR group) were systematically smaller compared with patients who did not achieve SVR (non-SVR group). These group differences were above the 0.2 threshold value that indicates a small group effect for all but 2 of the SF-36v2 scales: social functioning and role-emotional. The largest effect size (Cohen’s d = 0.56) was observed for the general health scale. For this scale, the mean change corresponded to an actual increase of 1.7 points in the SVR group and a decline of −3 points in the non-SVR group. Mean change scores experienced by both SVR and non-SVR groups were smaller than the recommended MIC of 7.2 for the general health scale. All SF-36v2 mean change scores were smaller than MIC estimates. Similarly, although effects for the CLDQ-HCV were >0.3 for all scales, the mean change scores were smaller than the MIC values in all cases.

### Logistic regression analyses: clinical factors as predictors of meaningful changes in HRQoL

**Fig. 3:**
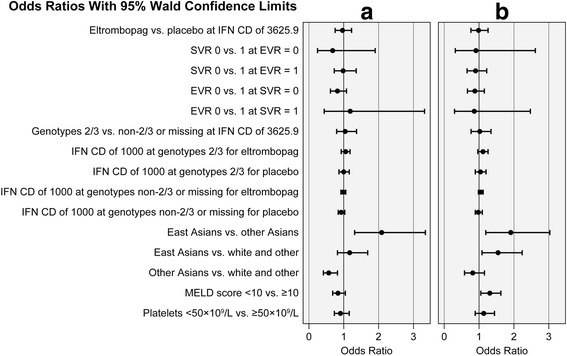
Odds ratios of changes in CLDQ-HCV scores at various time points. Odds of (**a**) a drop in CLDQ-HCV scores greater than the MIC at the end of AVT treatment and (**b**) a return to baseline levels in overall CLDQ-HCV score. AVT, antiviral therapy; CD, cumulative dose; CLDQ-HCV, Chronic Liver Disease Questionnaire–Hepatitis C Virus version; EVR, early virologic response; IFN, interferon; MELD, Model for End-Stage Liver Disease; MIC, minimally important change; SVR, sustained virologic response

Results of the logistic regression analysis during AVT (from AVT baseline to end of treatment) showed no OR differences in patients’ decrease in overall CLDQ-HCV score, relative to the MIC, for any of the 5 clinical factors or the specified interactions (Fig. 3a, goodness of fit is shown in Additional file 2). However, these results did show an ethnicity effect (*P* = 0.0025): East Asians were significantly more likely to report a meaningful drop in overall CLDQ-HCV score versus non-East Asians (OR 2.09 [95 % CI: 1.30, 3.34]), while non-East Asians were significantly less likely to report such a drop versus whites/other ethnicities (OR 0.56 [95 % CI: 0.39, 0.80]). Although model details suggest a good fit of the model to the data, the very small *R*^2^ value (0.02) and relatively small AUC (0.56) indicate that the group of variables included in the model is a poor predictor of whether a patient’s CLDQ-HCV score drops by a meaningful amount from study baseline to endpoint.

Analysis of return to AVT baseline and restoration of baseline HRQoL after completing 24 weeks of treatment are shown in Fig. 3b. None of the 5 clinical factors or viral response interaction terms was found to influence the ORs of patients’ return to their AVT baseline overall CLDQ-HCV score at the 24-week post-treatment follow-up. These results also showed an ethnicity effect (*P* = 0.0207): patients of East Asian ethnicity were significantly more likely to demonstrate a return to their baseline overall CLDQ-HCV score at the 24-week post-treatment follow-up versus non-East Asians (OR 1.89 [95 % CI: 1.19, 3.02]) or whites/other ethnicities (OR 1.54 [95 % CI: 1.07, 2.22]). Significant differences were also found regarding the MELD score (*P* = 0.02): patients with a MELD score <10 were significantly more likely to demonstrate a return to their baseline overall CLDQ-HCV score versus those with a score ≥10 at the 24-week post-treatment follow-up (OR 1.30 [95 % CI: 1.04, 1.62]). Although the model details support a good fit to the data, the very small *R*^*2*^ value (0.02) and relatively small area under the curve (AUC; 0.58) indicate that the group of variables included in the model is a poor predictor of whether a patient returns to his or her baseline overall CLDQ-HCV score.

**Fig. 4:**
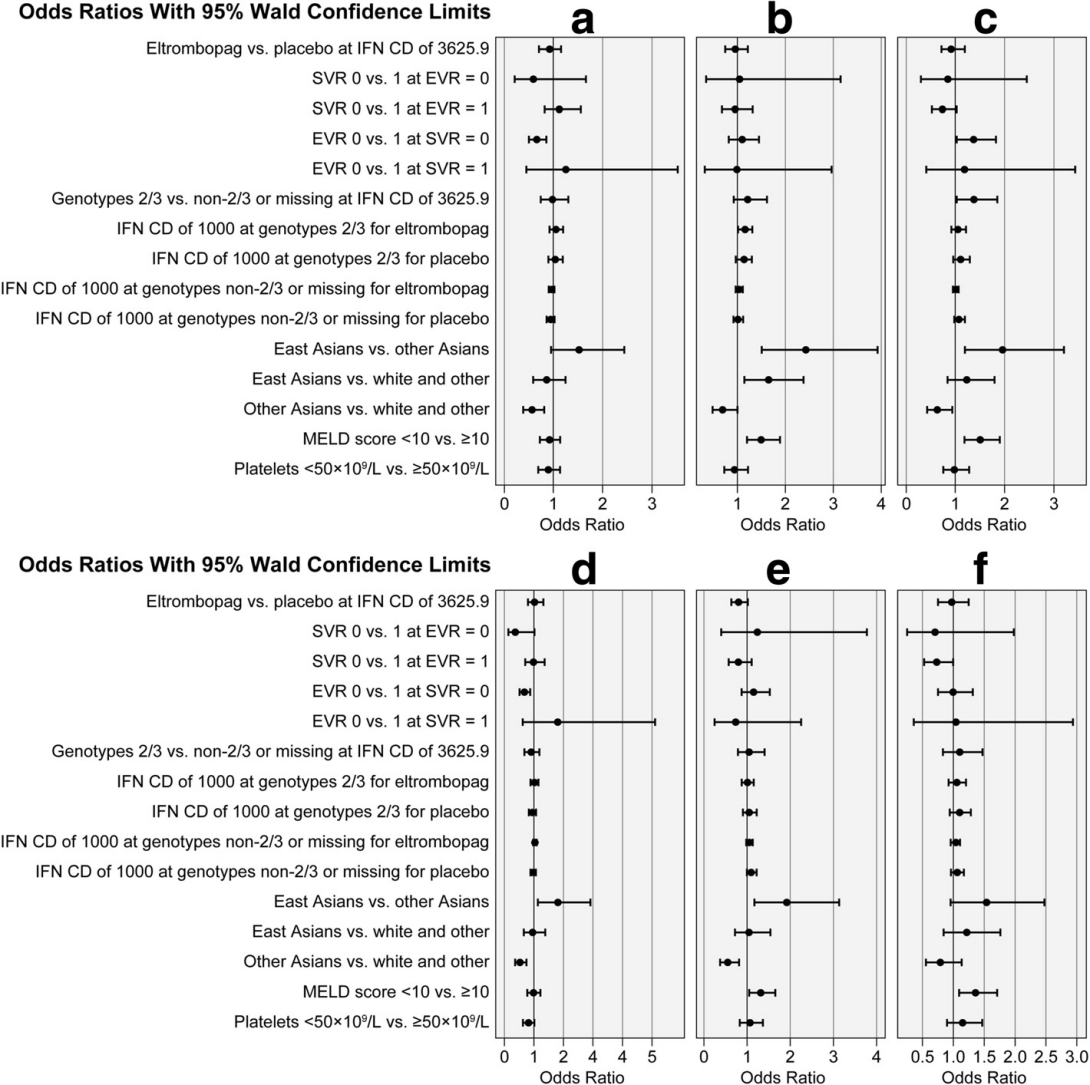
Odds ratios of changes in SF-36v2 PCS and MCS scores at various time points. Odds of (**a**) a drop in SF-36v2 PCS scores greater than the MIC at the end of AVT treatment; (**b**) a return to baseline levels in SF-36v2 PCS scores; (**c**) a return to baseline levels in SF-36v2 PCS (submission scoring) scores; (**d**) a drop in SF-36v2 MCS scores greater than the MIC at the end of AVT treatment; (**e**) a return to baseline levels in SF-36v2 MCS scores; and (**f**) a return to baseline levels in SF-36v2 MCS (submission scoring) scores. AVT, antiviral therapy; CD, cumulative dose; EVR, early virologic response; IFN, interferon; MELD, Model for End-Stage Liver Disease; MIC, minimally important change; SF-36v2, Short-Form 36 Health Survey version 2; SVR, sustained virologic response

Among the non-SVR group, a statistically significant decrease in the ORs of a meaningful drop in PCS scores relative to the MIC, from AVT baseline to the end of AVT, was found for patients who did not achieve EVR versus those who did (OR 0.65 [95 % CI: 0.49, 0.85]) (Fig. 4a). No significant differences in the ORs of demonstrating a meaningful drop in PCS scores from AVT baseline to the end of AVT were found between East Asians and other ethnicities (OR 0.85 [95 % CI: 0.59, 1.23]), although an overall race effect was demonstrated (*P* = 0.0064) due to non-East Asians being significantly less likely to experience such a drop versus whites/other ethnicities (OR 0.56 [95 % CI: 0.39, 0.80]).

At the 24-week post-treatment follow-up visit post-AVT, no differences were found in the ORs of patients’ return to their baseline SF-36v2 PCS score across any of the 5 clinical factors or the specified interactions (Fig. 4b; goodness of fit is shown in Additional file 2). The results show an ethnicity effect (*P* = 0.0017): East Asians were significantly more likely to demonstrate a return to baseline PCS score at the 24-week post-treatment follow-up versus non-East Asians (OR 2.41 [95 % CI: 1.48, 3.92]) or whites/other ethnicities (OR 1.64 [95 % CI: 1.13, 2.37]), while non-East Asians were significantly less likely to demonstrate a return versus whites/other ethnicities (OR 0.68 [95 % CI: 0.46, 0.99]). Significant differences were also found regarding the MELD score (*P* = 0.0009): patients with a MELD score <10 were significantly more likely to demonstrate a return to their baseline PCS score at the 24-week post-treatment follow-up versus those with a score ≥10 (OR 1.48 [95 % CI: 1.18, 1.87]). Again, model details suggest a good fit of the model to the data (Additional file 2); however, the very small *R*^*2*^ value (0.02) and relatively small AUC (0.58) indicate this group of variables is a poor predictor of whether a patient returns to their baseline PCS score.

Differences in the ORs of return to baseline SF-36v2 PCS (correlated physical and mental health factor model) scores at the 24-week post-treatment follow-up across some of the 5 clinical factors were also found (Fig. 4c). These differences between AVT baseline and the 24-week post-treatment follow-up included: statistically significantly greater odds for a return to baseline in PCS (correlated physical and mental health factor model) scores for patients who did not achieve EVR versus those who did (OR 1.36 [95 % CI: 1.01, 1.82]); statistically significantly larger odds of experiencing a return to AVT baseline for patients with a non-2/3 genotype (OR 1.37 [95 % CI: 1.02, 1.84]); East Asians were significantly more likely to demonstrate a return to their baseline PCS (correlated physical and mental health factor model) score versus non-East Asians (OR 1.936 [95 % CI: 1.18, 3.18]), and non-East Asians were also significantly less likely to demonstrate a return versus whites/other ethnicities (OR 0.63 [95 % CI: 0.43, 0.92]); and a significantly increased odds of patients with a MELD score <10 demonstrating a return to their baseline PCS (correlated physical and mental health factor model) score (OR 1.49 [95 % CI: 1.17, 1.89]).

Increased odds of a meaningful drop in MCS scores from AVT baseline to the end of AVT relative to the MIC were related to race (*P* = 0.0020), with East Asians more likely to experience such a drop relative to non-East Asians (OR 1.81 [95 % CI: 1.13, 2.90]); non-East Asians were also significantly less likely to demonstrate such a drop versus whites/other ethnicities (OR 0.525 [95 % CI: 0.37, 0.75]). Among the non-SVR group, patients who did not achieve EVR had a statistically significant decrease in the ORs of a drop in their MCS score that exceeded the MIC from baseline to the end of AVT versus patients who achieved EVR (OR 0.663 [95 % CI: 0.50, 0.87]) (Fig. 4d).

No differences in the ORs of patients’ return to baseline SF-36v2 MCS scores at the 24-week post-treatment follow-up were found across any of the 5 clinical factors or the specified interactions (Fig. 4e). Results show an ethnicity effect (*P* = 0.0072): East Asians were significantly more likely to demonstrate a return to their baseline MCS score at the 24-week post-treatment follow-up visit than were non-East Asians (OR 1.91 [95 % CI: 1.17, 3.14]), while non-East Asians were significantly less likely to demonstrate a return than were whites/other ethnicities (OR 0.550 [95 % CI: 0.37, 0.81]). Significant differences (*P* = 0.0183) were also found regarding the MELD score: patients with a MELD score <10 were significantly more likely to demonstrate a return to their baseline MCS score at the 24-week post-treatment follow-up visit versus patients with a MELD score ≥10 (OR 1.32 [95 % CI: 1.05, 1.66]).

No differences in the ORs of patients’ return to baseline SF-36v2 MCS (correlated physical and mental health factor model) scores at the 24-week post-treatment follow-up were found across any of the 5 clinical factors for the MCS scored using the alternate approach (Fig. 4f). However, patients with a MELD score <10 had significantly increased ORs of demonstrating a return to their baseline MCS (correlated physical and mental health factor model) score at the 24-week post-treatment follow-up (OR 1.36 [95 % CI: 1.08, 1.70]).

Again, among the non-SVR group, patients who did not achieve EVR (i.e. no viral response early or late) had a statistically significant lower odds of a drop in MCS (correlated factor model) score equal or greater than the MIC between AVT initiation and end of AVT compared with patients who achieved EVR (OR 0.76 [95 % CI: 0.58, 0.99]) (data not shown). Similarly, non-East Asians were also less likely to report a meaningful drop in MCS (correlated factor model) scores from AVT baseline to the 24-week post treatment follow-up relative to whites/other ethnicities (OR 0.54 [95 % CI: 0.38, 0.77]).

Results and model specifications in this article were updated from those originally submitted as part of regulatory files in order to reflect the most recent and preferred analysis algorithm from the developers of the original instruments, and further exploration and testing of goodness-of-fit criteria and underlying model econometric assumptions. Alternative models excluded specific clinical variables and/or interactions among clinical variables as described earlier, or otherwise mathematically transformed predictor variables. Tests of these alternate models did not result in fundamentally different conclusions being reached regarding the predictive variables compared with analysis using original models.

## Discussion

Analyses presented in this report indicate that the physical and mental functioning of the pooled sample from the ENABLE studies were below that of the general US population, although the differences were small (on the order of <0.5 SD) in most cases. SF-36 scores from previous publications [5, 9, 10] generally agree with those in this study, particularly regarding the impact upon specific SF-36v2 HRQoL domains. All patients enrolled in the ENABLE trials had thrombocytopenia, and 90 % had cirrhosis. Based on the similar safety and efficacy results of the individual ENABLE trials, the descriptive HRQoL analyses in the pooled sample is not expected to differ from HRQoL results analyzed separately.

An important finding of this study is the observed decline in HRQoL from baseline at AVT initiation and during the course of treatment, with a return to baseline levels upon completing treatment. Although declines in HRQoL were smaller in eltrombopag-treated patients, differences between this group and placebo-treated patients were generally too small to be interpreted as clinically meaningful when the change in HRQoL from baseline to the end of treatment and from AVT baseline to 24 weeks following the completion of AVT were assessed. Treatment with eltrombopag did not mitigate the effects of AVT on HRQoL or worsen adverse events or symptoms of AVT versus placebo. The data suggest that eltrombopag, by keeping platelet counts at levels sufficient to initiate, continue, and complete INF-based AVT, can provide patients who have severe liver disease an opportunity to receive IFN-based treatment without increasing adverse events such as flu-like symptoms or depression that would further impact their HRQoL. Although new non-IFN-based therapies are approved, these treatments may not be available to patients with HCV in all geographical regions of the world, where IFN may still be used to treat patients with HCV infection.

Regarding both EVR and SVR, analyses of HRQoL changes showed a greater association and clearer patterns in terms of smaller declines in mean HRQoL scores among patients achieving a virologic response. Nevertheless, mean changes in patients achieving EVR and SVR tended to be small compared with accepted thresholds of minimally (clinically) important difference. Furthermore, differences between patients achieving versus not achieving EVR were generally too small to be interpreted as meaningful. Although differences in effect sizes were slightly larger between patients who did and did not achieve SVR, these were also too small to be interpreted as meaningful. Achieving SVR and eliminating the viral infection would be anticipated to slow the progression of liver disease, but do not reverse it. Partitioning the HRQoL effects into those related to INF-based therapy versus those related to viral clearance (EVR and SVR), IFN-based treatment would likely account for the majority of the decline in HRQoL during treatment and improvement in HRQoL after stopping treatment.

Patients infected with HCV genotypes 2 and 3 received 24 weeks of AVT versus 48 weeks for patients infected with other genotypes. Nonetheless, with the exception of the small interaction between SVR and EVR, none of the 5 clinical factors (SVR, EVR, genotype [2/3 vs. non-2/3], treatment [eltrombopag or placebo], and cumulative IFN dose) was confirmed as a predictor of meaningful change in HRQoL. However, patients with a MELD score <10 (those with less severe cirrhosis and liver damage) were significantly more likely to return to their overall baseline CLDQ-HCV, PCS, and MCS scores.

The logistic regression models revealed consistent OR patterns for East Asians versus other Asians, and other Asians versus white/other ethnicities for the CLDQ-HCV and SF-36v2 PCS and MCS scores. East Asian patients were consistently more likely to experience a meaningful drop in HRQoL than white/other patients, but also more likely to experience a return to their baseline scores. Other Asians were generally less likely to experience a drop in HRQoL versus white/other patients. Of note, Asian patients received a reduced eltrombopag dose, although eltrombopag treatment seemed to have little impact on HRQoL changes versus placebo. Assessments of HRQoL in Asian patients with chronic HCV infections receiving interferon-based AVT have not been reported in the literature. However, cultural differences in perceptions regarding health status may contribute to differences in scores for the different scales of the SF-36v2, as was seen in a study in Taiwan that revealed the vitality scale was a stronger measure of mental health than physical health in the local population [31].

The current study has limitations. Data were derived from clinical trials, which may not fully reflect real-world clinical practice. Relatively few patients achieved SVR or EVR compared with those treated with AVT and randomized to receive eltrombopag; therefore, we may not have been able to detect robust average change in HRQoL because of the small number of responders. A general US population was used as the norm in these analyses; however, the trials were international, and differences seen in Asian patients enrolled in these studies may be affected by this. Additionally, there are many options available for measuring the MIC and no particular method is indicative of or reliable in reflecting how patients themselves may perceive change in their health status or quality of life. The choice to use one half SD as an estimate of the MIC was reasonable for expressing observed changes in a standardized way, but is also imperfect and may under- or overestimate the true MIC, as discussed by Farivar [32] in a critical review of Norman et al. [27].

## Conclusions

A decline in HRQoL was observed during AVT with a return to baseline levels at 24-week post treatment follow-up. Treatment with eltrombopag neither significantly reduced nor enhanced the pattern of changes in HRQoL associated with AVT. Clinical factors did not appear to have large effects on HRQoL, but East Asian patients were more likely to experience declines in HRQoL during AVT.

## Additional files

Additional file 1: Additional information on norm-based scoring and summary score calculations for the SF-36v2. (PDF 36 kb) Additional file 2: Goodness of fit for logistic regression models (Figs 3 and 4). (PDF 229 kb)

## Abbreviations

AIC: akaike information criterion
AUC: area under the curve
AVT: antiviral therapy
CI: confidence interval
CLDQ-HCV: Chronic Liver Disease Questionnaire–HCV version
EVR: early virologic response
HCV: hepatitis C virus
HRQoL: health-related quality of life
IFN: interferon
Log L: log of the likelihood function
MCS: mental component summary
MELD: Model for End-Stage Liver Disease
MIC: minimally important change
NBS: norm-based scoring
OR: odds ratio
PCS: physical component summary
PEG-IFN: pegylated interferon
RBV: ribavirin
SD: standard deviation
SF-36v2: Short-Form 36 Health Survey version 2
SVR: sustained virologic response
