# Supplementary material for: Health-related quality of life in thrombocytopenic patients with chronic hepatitis C with or without cirrhosis in the ENABLE-1 and ENABLE-2 studies
Source: Health Qual Life Outcomes. 2016 Mar 22;14:49. doi: 10.1186/s12955-016-0447-1 (PMC4802726; doi:10.1186/s12955-016-0447-1)
Supplement: Additional file 1: — Additional information on norm-based scoring and summary score calculations for the SF-36v2. (PDF 36 kb) [file 12955_2016_447_MOESM1_ESM.pdf]

Additional file 1

Additional information on norm-based scoring

Norm-based scoring (NBS) scale scores are calculated by first converting the raw scores onto a 0–100 metric. This scaling is achieved by adding coded responses to all items within a scale, subtracting the lowest possible total score for those items from that sum, dividing this number by the potential range of responses, and then multiplying this number by 100. As an example, the physical functioning (PF) scale is computed from responses to 10 items (PF01–PF10), with each of these items having 3 possible responses (1: Yes, limited a lot; 2: Yes, limited a little; and 3: No, not limited at all). Thus, the lowest possible total score for the sum of all PF items is 10, the highest possible total score is 30, and the range of the possible total score for this scale is 20. Calculation of the 0–100 PF scale score would thus use the following equation:

PF = 100*[((PF01+PF02+PF03+PF04+PF05+PF06+PF07+PF08+PF09+PF10)-10)/20].

The 0–100 scale scores are then transformed into standardized *z* scores using the mean and standard deviation (SD) found in the general US population norms for each scale. These *z* scores are then converted into T scores by multiplying each by 10 and adding 50. The general US population norms mean the Short-Form 36 Health Survey version 2 (SF-36v2) PF 0–100 score is 80.3 with an SD of 26.1. Derivation of the PF_NBS for a patient in the current study would therefore involve the following calculation: PF_NBS = 50 + 10*([PF- 80.3]/26.1), where PF is the patient's 0-100 PF score, calculated as described above. Physical and mental component summary scores are each calculated through weighted sums of all 8 scale *z* scores. A more detailed description of this scoring process can be found in the SF-36v2 manual [1].

Additional information on summary score calculations for the SF-36v2

The algorithm described by Farivar and colleagues [2] differs from the original SF-36v2 with regard to summary score calculation in that it uses a correlated (oblique) physical and mental health factor model in lieu of the uncorrelated (orthogonal) factor solution used in the original SF-36v2. The oblique factor solution results in a lower number of negative factor scoring coefficients than are seen if the standard orthogonal factor solution is used. Correlated physical and mental health scores were calculated by multiplying the *z* score for each SF-36v2 scale by the respective scoring coefficient from the obliquely

rotated 2-factor solution. These scores were then converted into T scores by multiplying each by 10 and adding 50.

References

1. QualityMetric. User's manual for the SF-36v2 Health Survey, 3rd ed. Lincoln, RI; 2011.

2. Farivar SS, Cunningham WE, Hays RD. Correlated physical and mental health summary scores for the SF-36 and SF-12 Health Survey, V.I. Health Qual Life Outcomes. 2007;5:54.
