# Supplementary material for: Health-related quality of life in thrombocytopenic patients with chronic hepatitis C with or without cirrhosis in the ENABLE-1 and ENABLE-2 studies
Source: Health Qual Life Outcomes. 2016 Mar 22;14:49. doi: 10.1186/s12955-016-0447-1 (PMC4802726; doi:10.1186/s12955-016-0447-1)
Supplement: Additional file 2: — Goodness of fit for logistic regression models (Figs 3 and 4). (PDF 229 kb) [file 12955_2016_447_MOESM2_ESM.pdf]

**Additional file 2. Goodness of fit for logistic regression models (Figures 3 and 4)**

| | **Model Fit Statistic** | | | | | | |
|---|---|---|---|---|---|---|---|
| | **Hosmer-Lemeshow $\chi^2$** | **AIC** | **SC** | **-2 Log L** | $R^2$ | **AUC** | ***P*** |
| **CLDQ-HCV scores (Figure 2)** | | | | | | | |
| Scores greater than the MIC during AVT | 3.81 | 1893.16 | 1961.46 | 1867.16 | 0.02 | 0.58 | 0.87 |
| Return to baseline levels in overall scores | 13.51 | 1936.08 | 2004.38 | 1910.08 | 0.02 | 0.56 | 0.10 |
| **SF36v2 PCS (Figure 3)** | | | | | | | |
| Return to baseline levels | 7.83 | 1790.27 | 1858.58 | 1764.27 | 0.02 | 0.58 | 0.45 |
| Drop in scores greater than the MIC during AVT | 8.60 | 1925.40 | 1993.71 | 1899.40 | 0.02 | 0.58 | 0.38 |
| Return to baseline levels (submission scoring) | 14.49 | 1743.37 | 1811.68 | 1717.37 | 0.02 | 0.58 | 0.07 |
| **SF36v2 MCS (Figure 3)** | | | | | | | |
| Return to baseline levels | 7.43 | 1823.24 | 1891.54 | 1797.24 | 0.02 | 0.58 | 0.49 |
| Drop in scores greater than the MIC during AVT | 9.75 | 1923.89 | 1992.19 | 1897.89 | 0.02 | 0.59 | 0.28 |
| Return to baseline levels (submission scoring) | 17.94 | 1852.18 | 1920.49 | 1826.18 | 0.02 | 0.58 | 0.02 |

AIC, Akaike information criterion; AUC, area under the curve; AVT, antiviral therapy; CLDQ-HCV, Chronic Liver Disease Questionnaire–Hepatitis C Virus version; Log L, log of the likelihood function; MIC, minimally important change; SC, Schwarz Criterion; SF-36v2, Short-Form 36 Health Survey version 2.

**References**


1. Giannini EG: **Review article: thrombocytopenia in chronic liver disease and pharmacologic treatment options.** *Aliment Pharmacol Ther* 2006, **23:**1055-1065.
2. Schmid M, Kreil A, Jessner W, Homoncik M, Datz C, Gangl A, Ferenci P, Peck-Radosavljevic M: **Suppression of haematopoiesis during therapy of chronic hepatitis C with different interferon alpha mono and combination therapy regimens.** *Gut* 2005, **54:**1014-1020.
3. Ganser A, Carlo-Stella C, Greher J, Volkers B, Hoelzer D: **Effect of recombinant interferons alpha and gamma on human bone marrow-derived megakaryocytic progenitor cells.** *Blood* 1987, **70:**1173-1179.
4. Wang Q, Miyakawa Y, Fox N, Kaushansky K: **Interferon-alpha directly represses megakaryopoiesis by inhibiting thrombopoietin-induced signaling through induction of SOCS-1.** *Blood* 2000, **96:**2093-2099.
5. Bjornsson E, Verbaan H, Oksanen A, Fryden A, Johansson J, Friberg S, Dalgard O, Kalaitzakis E: **Health-related quality of life in patients with different stages of liver disease induced by hepatitis C.** *Scand J Gastroenterol* 2009, **44:**878-887.
6. Dan AA, Martin LM, Crone C, Ong JP, Farmer DW, Wise T, Robbins SC, Younossi ZM: **Depression, anemia and health-related quality of life in chronic hepatitis C.** *J Hepatol* 2006, **44:**491-498.
7. Ware JE, Jr., Bayliss MS, Mannocchia M, Davis GL: **Health-related quality of life in chronic hepatitis C: impact of disease and treatment response. The Interventional Therapy Group.** *Hepatology* 1999, **30:**550-555.
8. McHutchison JG, Ware JE, Jr., Bayliss MS, Pianko S, Albrecht JK, Cort S, Yang I, Neary MP, Hepatitis Interventional Therapy G: **The effects of interferon alpha-2b in combination with ribavirin on health related quality of life and work productivity.** *J Hepatol* 2001, **34:**140-147.
9. Bonkovsky HL, Woolley JM: **Reduction of health-related quality of life in chronic hepatitis C and improvement with interferon therapy. The Consensus Interferon Study Group.** *Hepatology* 1999, **29:**264-270.

10. Bonkovsky HL, Snow KK, Malet PF, Back-Madruga C, Fontana RJ, Sterling RK, Kulig CC, Di Bisceglie AM, Morgan TR, Dienstag JL, et al: **Health-related quality of life in patients with chronic hepatitis C and advanced fibrosis.** *J Hepatol* 2007, **46:**420-431.

11. Neary MP, Cort S, Bayliss MS, Ware JE, Jr.: **Sustained virologic response is associated with improved health-related quality of life in relapsed chronic hepatitis C patients.** *Semin Liver Dis* 1999, **19 Suppl 1:**77-85.

12. Afdhal NH, Dusheiko GM, Giannini EG, Chen PJ, Han KH, Mohsin A, Rodriguez-Torres M, Rugina S, Bakulin I, Lawitz E, et al: **Eltrombopag increases platelet numbers in thrombocytopenic patients with HCV infection and cirrhosis, allowing for effective antiviral therapy.** *Gastroenterology* 2014, **146:**442-452 e441.

13. Maruish ME. QualityMetric: *User's manual for the SF-36v2 Health Survey.* 3rd edn. Lincoln, RI; 2011.

14. Younossi ZM, Guyatt G, Kiwi M, Boparai N, King D: **Development of a disease specific questionnaire to measure health related quality of life in patients with chronic liver disease.** *Gut* 1999, **45:**295-300.

15. Two R, Verjee-Lorenz A, Clayson D, Dalal M, Grotzinger K, Younossi ZM: **A methodology for successfully producing global translations of patient reported outcome measures for use in multiple countries.** *Value Health* 2010, **13:**128-131.

16. Benini F, Distefano L, Baisini O, Pigozzi MG, Lanzini A: **Efficacy and tolerability of combination therapy with interferon-alfa plus ribavirin in patients with chronic hepatitis C virus infection: a single-center study in relapsers and nonresponders to previous treatment with high-dose interferon-alfa monotherapy.** *Curr Ther Res Clin Exp* 2003, **64:**140-150.

17. Witthoft T, Moller B, Wiedmann KH, Mauss S, Link R, Lohmeyer J, Lafrenz M, Gelbmann CM, Huppe D, Niederau C, Alshuth U: **Safety, tolerability and efficacy of peginterferon alpha-2a and ribavirin in chronic hepatitis C in clinical practice: The German Open Safety Trial.** *J Viral Hepat* 2007, **14:**788-796.

18. Chang CH, Chen KY, Lai MY, Chan KA: **Meta-analysis: ribavirin-induced haemolytic anaemia in patients with chronic hepatitis C.** *Aliment Pharmacol Ther* 2002, **16:**1623-1632.

19. Conversano C, Carmassi C, Carlini M, Casu G, Gremigni P, Dell'Osso L: **Interferon alpha Therapy in Patients with Chronic Hepatitis C Infection: Quality of Life and Depression.** *Hematol Rep* 2015, **7:**5632.

20. Lin FC, Young HA: **Interferons: Success in anti-viral immunotherapy.** *Cytokine Growth Factor Rev* 2014, **25:**369-376.

21. Arora S, O'Brien C, Zeuzem S, Shiffman ML, Diago M, Tran A, Pockros PJ, Reindollar RW, Gane E, Patel K, et al: **Treatment of chronic hepatitis C patients with persistently normal alanine aminotransferase levels with the combination of peginterferon alpha-2a (40 kDa) plus ribavirin: impact on health-related quality of life.** *J Gastroenterol Hepatol* 2006, **21:**406-412.

22. John-Baptiste AA, Tomlinson G, Hsu PC, Krajden M, Heathcote EJ, Laporte A, Yoshida EM, Anderson FH, Krahn MD: **Sustained responders have better quality of life and productivity compared with treatment failures long after antiviral therapy for hepatitis C.** *Am J Gastroenterol* 2009, **104:**2439-2448.

23. Ghany MG, Strader DB, Thomas DL, Seeff LB, American Association for the Study of Liver D: **Diagnosis, management, and treatment of hepatitis C: an update.** *Hepatology* 2009, **49:**1335-1374.

24. Spiegel BM, Younossi ZM, Hays RD, Revicki D, Robbins S, Kanwal F: **Impact of hepatitis C on health related quality of life: a systematic review and quantitative assessment.** *Hepatology* 2005, **41:**790-800.

25. Farivar SS, Cunningham WE, Hays RD: **Correlated physical and mental health summary scores for the SF-36 and SF-12 Health Survey, V.I.** *Health Qual Life Outcomes* 2007, **5:**54.

26. Cohen J: *Statistical Power Analysis for the Behavioral Sciences.* 2 edn. New York, NY: Taylor & Francis; 1988.

27. Norman GR, Sloan JA, Wyrwich KW: **Interpretation of changes in health-related quality of life: the remarkable universality of half a standard deviation.** *Med Care* 2003, **41:**582-592.

28. **EASL International Consensus Conference on Hepatitis C. Paris, 26-28, February 1999, Consensus Statement. European Association for the Study of the Liver.** *J Hepatol* 1999, **30:**956-961.

29. McHutchison JG, Manns M, Patel K, Poynard T, Lindsay KL, Trepo C, Dienstag J, Lee WM, Mak C, Garaud JJ, et al: **Adherence to combination therapy enhances sustained response in genotype-1-infected patients with chronic hepatitis C.** *Gastroenterology* 2002, **123:**1061-1069.

30. Hosmer S, Lemeshow S: *Applied Logistic Regression.* New York: Wiley and Sons; 1989.

31. Tseng HM, Lu JF, Gandek B: **Cultural issues in using the SF-36 Health Survey in Asia: results from Taiwan.** *Health Qual Life Outcomes* 2003, **1:**72.

32. Farivar SS, Liu H, Hays RD: **Half standard deviation estimate of the minimally important difference in HRQOL scores?** *Expert Rev Pharmacoecon Outcomes Res* 2004, **4:**515-523.
